# Measuring moral distress in Swedish maternal and neonatal healthcare: validation of an adapted MDS‑R and development of a criterion‑based index

**DOI:** 10.1038/s41598-026-52337-6

**Published:** 2026-05-12

**Authors:** Magnus Akerstrom, Karolina Linden, Emina Hadžibajramović

**Affiliations:** 1 Institute of Stress Medicine, Region Västra Götaland, Gothenburg, Sweden; 2 School of Public Health and Community Medicine, Institute of Medicine, Sahlgrenska Academy, University of Gothenburg, Gothenburg, Sweden; 3 Institute of Health and Care Sciences, Sahlgrenska Academy, University of Gothenburg, Gothenburg, Sweden

**Keywords:** Moral distress scale revised (MDS-R), Content validity, Face validity, Construct validity, Maternal and neonatal healthcare workers, Criterion-based index, Health care, Health occupations, Medical research, Psychology, Psychology

## Abstract

There is a lack of methods for quantifying moral distress which limits the possibility to address this emerging challenge in practice. The aims were to investigate the content and face validity of an adapted version of the Moral Distress Scale Reversed (MDS-R) for use in Swedish maternal and neonatal health care, to develop a criterion-based index for the identification of high moral distress, and to assess its relationship with stress, burnout complaints, intention to leave and job satisfaction. The content and face validity were assessed using expert assessments (*n* = 10) and cognitive interviews with experienced professionals (*n* = 5). A global moral distress index was developed using a criterion-based approach and survey data from maternal and neonatal healthcare workers (*n* = 951) within the longitudinal COPE Staff cohort were used for construct validity. Results showed good content and face validity. 28% reported high moral distress which was associated with increased levels of stress, burnout complaints and intention to leave, and with decreased levels of job satisfaction (*p* < 0.001, respectively), indicating good construct validity. Thus, the Swedish maternal and neonatal version of the MDS-R may be used to systematically identify those at risk of adverse effects due to high moral distress, enabling early intervention and prevention.

**Supplementary Information:**

The online version contains supplementary material available at 10.1038/s41598-026-52337-6.

## Introduction

Moral distress can be described as a negative reaction to a morally challenging situation and has been associated with adverse outcomes for healthcare workers, patients and healthcare organisations^1^. The maternal and neonatal healthcare sector is no exception, and moral distress has increasingly been recognised as an emerging challenge^2,3^ affecting both job satisfaction and levels of stress, burnout complaints and staff turnover^1,4–6^. Consequently, an increased understanding of the occurrence and prevention of moral distress within maternal and neonatal healthcare is urgently needed.

Moral distress was originally described by Jameton^7^ as arising when one knows the right thing to do, but perceived or prevailing institutional constrains prevent the “right” action. This definition has, over the years, been broadened to also include a range of morally undesirable events such as dilemmas, uncertainties and conflicts^8,9^. However, this expansion has been criticised for introducing conceptual ambiguities that may compromise the validity of empirical research. Such ambiguities may result in an overestimation of the prevalence of moral distress and limit our understanding of the root causes, which in turn may reduce the effectiveness of interventions aimed at decreasing moral distress within healthcare^10,11^. In particular, existing definitions and their corresponding measures of moral distress, present three challenges. Firstly, there is a need to distinguish between morally challenging situations (stressors) that an individual encounters as a natural part of clinical practice and the psychological and physiological reactions that arise when such challenges lack adequate solutions or support (i.e. moral distress)^9,10^. Secondly, measures also need to distinguish between moral distress caused by morally challenging situations, such as a high workload which compromises patient safety or quality of care, and other types of distress caused by challenging work-related situations such as the high workload itself^11^. Lastly, the use of indexes calculated as a summarised scores of the intensity and frequency of several morally challenging situations may further complicate the interpretation of the measurement of total moral distress^11,12^.

Many instruments for measuring moral distress have been developed to address the challenges above with the aim of providing reliable and valid measurements of this complex phenomenon, all with their pros and cons^12^. In this study, the Moral Distress Scale – Revised (MDS-R) was chosen as an instrument for quantitative measurement of moral distress because it focuses on morally challenging situations rather than on the physiological reactions to such situations, and it distinguishes between morally challenging and other challenging situations, aligning with the conceptual concerns outlined above. The instrument originates from the Moral Distress Scale (MDS) which was designed to measure moral distress among critical care nurses in the United States, focusing on the root causes of moral distress^13^. It was later revised and shortened to create the MDS-R designed for use in multiple healthcare settings and disciplines within the United States^14^. The MDS-R covers morally challenging situations (stressors) that are relevant in clinical settings (e.g. lack of continuity of care), internal constraints (e.g. lack of knowledge of alternative treatment plans) and external constraints (e.g. inadequate staffing) and measures the intensity (how would this stressor affect you) and the frequency (how often have you experienced such stressors) separately to facilitate a fuller understanding^14^.

When instruments are modified or used in new contexts, empirical evaluation is essential to ensure their validity and usability. Translation and cultural adaptation must be supported by validation to confirm suitability^15^. In the Swedish context, the MDS-R had previously been translated, culturally adapted, and validated for paediatric care^16^. In the present study, that Swedish paediatric version served as the basis for further adaptation to maternal and neonatal healthcare through contextual rewording and item reduction. To our knowledge, the MDS-R has not been adapted or validated for Swedish maternal and neonatal healthcare, where it aims to support understanding and prevention of this emerging yet complex phenomenon. When doing so, an increased focus on the construct´s measurement models has been suggested, especially for formative measurement models which have often been misspecified in the past^17^. Reflective measurement models assume the latent construct causes the indicators, so variations in the construct lead to variations in item scores (e.g., high fatigue yields high fatigue-item scores), whereas formative measurement models assume indicators collectively define the construct (e.g., socioeconomic status is formed by income, education, and occupation).

In addition, to effectively identify individuals experiencing high levels of moral distress, a global index is needed. However, aggregating moral stressors that vary in intensity and frequency poses methodological challenges and may reduce the accuracy of identifying those most affected^11^. Moreover, because formative models do not require unidimensionality, averaging or summing item responses is inappropriate, as indicators represent distinct aspects rather than manifestations of a single latent trait.

Thus, a criterion-based approach (CBA)^18^, where the theoretical knowledge of the experts in a particular field of interest is used, may be one way forward in developing an index for high moral distress. Such an index would enhance the usability of the MDS-R by providing a clearer overview of levels of high moral distress. A CBA relies on predefined response patterns that experts judge to reflect meaningful levels of the construct in question. In the context of formative measurement models, where items represent distinct contributors rather than interchangeable indicators, CBA provides a structured way to define how combinations of high intensity and non rare frequency across several morally challenging situations should be interpreted. Instead of relying on aggregated mean scores (which assume unidimensionality), CBA uses expert knowledge to specify which intensity–frequency combinations constitute “high” moral distress and how many such situations are required to indicate an accumulated burden. This makes the resulting index more transparent, theoretically anchored, and aligned with the formative nature of the MDS-R items.

Therefore, the aims of this study were to (1) assess the content and face validity of an adapted version of the Moral Distress Scale Revised (MDS-R) for use in the Swedish maternity and neonatal health care, (2) develop a global moral distress index that integrates both intensity and frequency of morally challenging situations, using a criterion-based approach, and, **(**3) evaluate the MDS-R in relation to job satisfaction, stress, burnout complaints and intention to leave as an aspect of construct validity.

## Methods

The present study was conducted using both qualitative and quantitative methods. In short, an adapted version of the Moral Distress Scale - Revised (MDS-R)^14^ was developed and validated for use in Swedish maternity and neonatal healthcare. This was done by assessing content and face validity^19^, constructing a global index for high moral distress using a criterion-based approach (CBA)^18^ and by evaluating construct validity in relation to job satisfaction, stress, burnout complaints, and in intention to leave.

### The adaption process of the MDS-R for use in the maternal and neonatal healthcare

The adaptation pathway comprised three sequential stages. First, the original U.S. MDS-R14 was previously translated, culturally adapted, and validated for Swedish paediatric care by af Sandeberg et al.^16^. Second, that Swedish paediatric version served as the source instrument for the present maternal and neonatal adaptation. Third, the 26 items of the Swedish paediatric version were reviewed for relevance to maternal and neonatal care, after which 10 items were retained and linguistically/contextually adapted for use across these settings.

In the MDS-R, morally challenging situations are phrased as statements, and the respondents are asked to indicate on a 0–4 Likert scale the disturbance intensity, i.e. how this situation would affect you, (on a scale ranging from *not at all* to *very negatively*) and the frequency, i.e. how often the situation arises, on a scale ranging from *never* to *very often*. While the original MDS-R^14^ comprises 21 items, the Swedish paediatric version includes an additional five items, resulting in 26 items covering various situations in which optimal care is endangered due to lack of resources (time, budget, competence), continuity of care or communication within the team, and making decisions with limited knowledge, etc.

The adaptation process to ensure content and face validity for the maternal and neonatal healthcare version was conducted in two steps. First, all 26 items of the Swedish version were critically assessed by the research team to determine their relevance and applicability to maternity and neonatal care settings. No formal domain matrix was predefined a priori for item selection. However, the assessment was guided by the study’s conceptual understanding of moral distress as a negative response to morally challenging situations, and the selection aimed to retain conceptual breadth across the main types of situations represented in the source instrument and considered pertinent to maternal and neonatal healthcare. Through iterative discussions, 10 items were identified as capturing morally distressing situations pertinent to professionals working within the maternity and neonatal care context. In practice, this meant prioritising items judged to represent important and recurring sources of moral distress in these settings, including situations related to resource constraints, lack of continuity or communication, decision-making under limited knowledge, and morally challenging care expectations or unresolved moral concerns. The intention was not to preserve all item categories in fixed proportions, but to retain a conceptually broad set of clinically meaningful situations relevant to the context. Second, the selected items were linguistically and contextually adapted to ensure their suitability across both maternity and neonatal care. This involved modifying the phrasing to reflect the realities and professional roles specific to the new settings, while preserving the original constructs. Accordingly, the present study evaluated the validity of the adapted Swedish maternal and neonatal version, rather than repeating the initial translation and cultural adaptation of the original instrument. The resulting 10 items of the Swedish maternity and neonatal healthcare version of the MDS-R questionnaire can be found in Table 2 in the Results section.

Lastly, the formative measurement model of the adapted maternal and neonatal healthcare version of the MDS-R was verified using the checklist provided by Fleuren et al.^17^, which consist of six objective criteria to determine whether a construct has a reflective or formative measurement model.

### Assessing the content and face validity

The content validity of the maternal and neonatal version of the MDS-R, i.e. how well a questionnaire covers all relevant aspects of the concept it aims to measure, was assessed in two steps. Firstly, a purposive sample of professionals (*n* = 4) including an obstetrician, a neonatologist, a midwife, and a nurse evaluated the clarity, relevance, and acceptability of the adapted items in the target contexts. Secondly, the lead author of the original Swedish adaptation^16^ was consulted to confirm that the modifications were appropriate and in line with the intended use of the scale.

The face validity, i.e., whether items appear to measure what they are supposed to measure, was also assessed in two steps. Firstly, a second expert group was formed, containing six researchers with thorough knowledge in work environment research, survey development and the maternal and neonatal healthcare. The group reviewed the items individually and the face validity was then assessed through iterative discussions within the group until agreement^19^.

Secondly, experienced maternal and neonatal healthcare workers were purposively invited to participate in this study with the goal of achieving a high information power regarding the research question^20^. Five maternal and neonatal healthcare workers from two different organisations (3 midwives and 2 physicians), with between 1 and 36 years of experience in the profession, participated after giving their informed consent. Structured cognitive interviews^21^, lasting between 17 and 76 min, were conducted, in which the participants were asked to think aloud while reviewing the adapted MDS-R and responding to the questions developed in the interview guide, Supplementary Information 1 – Interview guide cognitive interviews. The interviews were audio-taped and transcribed verbatim. The transcripts were coded and analysed using qualitative deductive content analysis^22^ based on whether items appear to measure what they are supposed to measure^19^.

### Development of a global criterion-based index

In this study, moral distress was conceptualised as the respondent’s negative/disturbing reaction to morally challenging situations rather than mere exposure to such situations. The adapted MDS-R was treated as a formative measurement model in which items capture distinct morally challenging situations constituting different contributors to the overall burden. From this perspective, “high” moral distress should reflect both (i) sufficiently high intensity in relation to a situation that occurs more than rarely, and (ii) a breadth across several morally challenging situations, rather than a single isolated experience. After assessing the content and face validity, a global index to identify individuals with a high moral distress was developed by the second expert group, containing six researchers with a thorough knowledge of work environment research, survey development, and maternal and neonatal healthcare, using a criterion-based approach (CBA) in line with the recommendations for handling formative measurement models^17^. CBA is based on the theoretical knowledge of the experts in a particular field of interest, and scores (in the present study, high and not high moral distress) are defined based on the frequency distribution of item responses in predefined response combinations (CBA criteria)^18^. Because moral distress was conceptualised as the disturbing impact of morally challenging situations, rather than mere exposure to them, both intensity and frequency were considered important in the classification of high moral distress. The classification process for deriving these criteria was conducted in four distinct phases. Firstly, all members of the expert group, described above, made their individual classifications of CBA criteria, i.e. individual assessments of what response options on the 0 to 4 scale corresponded to high intensity and high frequency of morally challenging situations, and the number of ratings with a high intensity and high frequency for the 10 items that corresponded to high moral distress. Secondly, these individual CBA criteria were compared and discussed, and final CBA criteria were developed through iterative discussions within the expert group until agreement. Thirdly, the proposed CBA criteria from the expert group were compared with the results from the cognitive interviews conducted with experienced maternal and neonatal healthcare workers. Lastly, the proportion of maternity and neonatal healthcare workers experiencing high moral distress was calculated and compared with *prior* estimates made by the expert group and with responses from the interviewed maternal and neonatal healthcare workers.

### Evaluating the construct validity

To investigate the construct validity aspect of the instrument, the relationship between the criterion-based index for high moral distress assessed with the adapted version of the MDS-R and job satisfaction, stress, burnout, and intention to leave was assessed. Since moral distress was treated as a formative construct without a continuous latent score, the global criterion‑based index, indicating high and not high moral distress was used. Job satisfaction, stress, burnout, and intention to leave were chosen based on previous knowledge and it was hypothesised that a high level of moral distress would be associated with high levels of stress, burnout complaints, and turnover intentions, and with low levels of job satisfaction^1,4–6,23^. The survey data were part of the longitudinal COPE Staff cohort and were collected between February and March 2023. The COPE Staff cohort has been thoroughly described previously^24^. In short, midwives, physicians, registered and assistant nurses within the Swedish maternal and neonatal healthcare were invited to complete an electronic questionnaire. This questionnaire was based on validated tools, including the adapted version of the MDS-R for maternal and neonatal healthcare workers, the Copenhagen Psychosocial Questionnaire (COPSOQ)^25^, and the Burnout Assessment Tool (BAT)^26^ used in this study. The participants took part after giving their informed consent, and a total of 951 maternal and neonatal healthcare workers participated in the 2023 survey wave. Demographic characteristics of the study participants in 2023 can be found in Table 1.

**Table 1:** Demographic characteristics of the study participants.

Demographics	Descriptives
All, n (%)	951 (100)
Profession, n (%)	
Midwife	441 (46)
Physician	303 (32)
Registered nurse	85 (9)
Assistant nurse	122 (13)
Age, mean (Q25 - Q75)	46.1 (37–55)
Type of care, n (%)	
Primary care	201 (21)
Hospital-based care	750 (79)

Job satisfaction and stress were measured using the Swedish version of the COPSOQ III questionnaire, where four items (e.g. “*How pleased are you with your work prospects?*”) form the job satisfaction dimension, and 3 items (e.g. “*How often have you had problems relaxing?*”) form the stress dimension. The Likert scale responses were converted to numbers, and indexes were computed as means of items ranging from 0 to 100^25^. Burnout was measured using the Burnout Assessment Tool (BAT), consisting of 23 items with five response categories, and the total burnout score was calculated as a mean value of all response with range 1–5 (lowest to highest)^26^. Respondents’ mean scores were then divided into three groups (no, mild and severe burnout complaints) using clinical cut-off values for BAT derived from the Swedish context^27^. Intention to leave was measured using a single item from the COPSOQ III questionnaire (“*How often do you consider looking for work elsewhere?*”) with the response options “*Always*,* Often*,* Sometimes*,* Rarely*,* and Never”*^25^.

The construct validity was assessed by investigating differences in the level of job satisfaction, stress, burnout complaints, and intention to leave between individuals with and without high moral distress using the chi-square test (for dichotomous variables), the Kruskal-Wallis test (for categorical variables with > 2 categories), and the Mann-Whitney U test (for continuous variables). Non‑parametric tests were applied because the analysed variables were ordinal and did not meet the assumptions required for parametric analyses, such as normally distributed residuals and equal intervals between response categories. Effect sizes were calculated according to Fiel Peres^28^ with │r│≥0.1, ≥ 0.3 and ≥ 0.5 and ŋ^2^ ≥0.01, ≥ 0.06 and ≥ 0.14, indicating a small, medium and large effect size. All analyses were conducted using SAS version 9.4; SAS Institute, Cary, NC, USA. P-values < 0.05 were considered statistically significant and no correction for multiple comparisons was applied.

## Results

### Content and face validity of the Swedish maternal and neonatal version of MDS-R

The content validity was assessed by healthcare professionals and through consultation with the lead author of the Swedish adaptation and validation of paediatric care, who confirmed that the Swedish maternal and neonatal version of the MDS-R broadly represented the domain of morally challenging situations that healthcare workers may face.

In the assessment of face validity, all items were regarded, both in the interviews and by the expert assessment, as relevant in relation to moral distress as well as linguistically and contextually satisfactory. This was further evident in that the interviewed maternal and neonatal healthcare workers could operationalise all items into real-life experiences of moral distress, Table 2. The items were also regarded as sufficient to capture moral distress, and no additional morally challenging situations were suggested.

**Table 2:** Quotes from interviews with maternal and neonatal healthcare workers, illustrating how they operationalised the different items.

Number	Items	Quotes
1	Being unable to provide the best possible care due to pressure from management to cut costs.	*“It’s often when someone from higher up*,* who isn’t on the care floor*,* tells us how to do the job. But it’s usually not about what’s best for the patient—it’s just about saving time.”*
2	Being expected to care for patients whom you feel you lack sufficient competence to care for.	*“Sometimes it happens that just because someone has a belly*,* we admit her to the maternity ward. It could be pneumonia. It could be medical patients. It could be surgical patients. If the ICU is full—even though they really need monitoring—suddenly it’s just*,* ‘Can’t you take care of this?’ It actually happens quite often.”*
3	Not addressing a moral issue, you have identified because the involved staff or management request that you do nothing.	*“And how would this situation affect me? Very negatively*,* I think*,* if it’s a patient. Because personally*,* I’m often very convinced. Of course*,* I take in what others say and think*,* but most of the time I’m quite confident in what the right course of action is.”*
4	Working with healthcare personnel who lack the necessary competence for the care of the mother or child.	*“No*,* I just get a picture like this and I think*,* dear God*,* what if I’m standing there with a very sick baby and there’s no neonatologist who knows what to do. I mean*,* that’s not fun.”*
5	Seeing the quality of patient care suffer due to poor communication within the team.	*“Yes*,* I can see it happening in the birthing unit*,* for example. It’s happened a few times at work that the coordinators haven’t really talked to the newer staff—their newer colleague—but instead just gone into her chart and written something like ‘You should do this…’ I’ve tried talking to my colleague*,* and she just hasn’t listened*,* basically.”*
6	Seeing patient care suffer due to lack of continuity, with many different caregivers involved.	*“It’s like when someone is admitted to the maternity ward and ends up having to switch staff. They might be there for 2–3 days*,* or even four*,* because of a severe infection. …… That can happen too*,* and I think it has an impact.”*
7	Working in a staffing situation (number/competence level) that you perceive as unsafe.	*“And with certain shifts now*,* you kind of feel like—before the shift starts—you think a lot: if there’s a lot to do*,* how will I manage? How can I support everyone in the best way? The patients—what do we do if someone gets sick? If we get a neonatal transport? I mean*,* yeah*,* lots of different scenarios running through your head. So yes*,* it takes a lot out of you*,* absolutely.”*
8	Not having time to conduct conversations with patients and families in the way you believe they should be conducted.	*“Yes*,* but it’s also one of those things—you can feel really overwhelmed*,* like if you have too many conversations to handle in one evening*,* for example*,* when you’re on the maternity ward. Then it’s incredibly stressful knowing that they’re not getting what they need.”*
9	Parents having unrealistic expectations of the care.	*“Are you kidding? Oh*,* now you want to sleep just because the baby has arrived. No*,* I’m terribly sorry—I’m not your nanny.”*
10	Making decisions about care/treatment when you are uncertain about what is right.	*“I think that’s really tough*,* especially when you’re relatively new. ……. Because*,* well*,* maternity care is sometimes… a judgment call. You don’t always know*,* but you have to go with the likelihood that this is what we believe is right.”*

Although all items were regarded as relevant, the interpretation of two items was discussed both in the interviews and in the expert group assessment. Item number 3 (i.e. *“Not addressing a moral issue*,* you have identified because the involved staff or management request that you do nothing”)* contains an element of negligence from staff or management, which many pointed out may be two completely different situations with different levels of intensity.

*“I would separate staff from management. Because staff*,* to me*,* are my coworkers.*

*I don’t have the same kind of relationship with management at all.”*

*                                           -Midwife, 28 years work experience, interview 1*

Also, item number 9 (i.e. *“Parents having unrealistic expectations of the care.”)* raised some discussion about the root cause of these expectations, which might also affect the interpretation of the results.

*“Yes*,* that’s a statement. I think healthcare staff sometimes think that way*,

*but you also need to be a bit more understanding. Because these unrealistic expectations*

*—where do they come from? They can be rooted in fears*,* wishes*,* and other things.”*

*                                           -Midwife, 20 years work experience, interview 2*

*“Yes*,* but well*,* I can think about it in two ways — it could also be about information*,

*that they are not well-informed and that they may be engaged or knowledgeable in different ways*.

*but still have unrealistic expectations.”*

*                                           -Physician, 27 years work experience, interview 4*

The perceived intensity and frequency of morally challenging situations was also believed to be affected by the level of experience within the profession:

*"Well*,* I’ve been thinking a bit… I’ve been thinking about this question. I believe it can be interpreted differently depending on whether you’re new or experienced in the field. Because if you’re new*,* you’re more sensitive when things don’t go well."*

*                                                                                      -Midwife, 28 years work experience, interview 1*

Although items 3 and 9 may potentially be interpreted differently by different respondents, the inclusion of both intensity and frequency ratings was considered by the respondents to help reduce potential bias in the quantitative measurement of moral distress. Similarly, the rating of both intensity and frequency of morally challenging situations was also believed to limit potential bias related to different interpretations of the items depending on whether the respondent was new or experienced in the profession.

### A criterion-based index for high moral distress

Both the expert group and the interviewed professionals assessed all 10 items as relevant to include in a global index, and the same criteria were decided to be applied to all items. They also stressed the importance of combining the quantitative measurements of both the intensity and frequency when developing an index for high moral distress.

*“Both are relevant*,* and I don’t think you could have excluded one of them*,

*because then you wouldn’t get the full picture.”*

*                                                                                      -Midwife, 28 years work experience, interview 1*

Since morally challenging situations are believed to be an inevitable part of maternal and neonatal healthcare, and strategies to handle such situations were both part of the training and developed through experience, only situations with an intensity of 3 or 4 (on a 0–4 scale) were regarded as contributing to high moral distress, even if they do not occur frequently.

*“It must be more important how it affects you than how often.*

*Although*,* of course*,* the two are connected.”*

*                                                                                      -Midwife, 36 years work experience, interview 3*

Also, a combination of several morally challenging situations with this intensity was assessed to be necessary to be classified as having high moral distress. Therefore, the criterion-based index combined intensity-frequency response patterns to capture high disturbance that is not rare (criterion A) and a multi-item threshold to capture accumulated burden across morally challenging situations (criterion B).

High moral distress was defined as: (A) reporting an intensity of 4 in combination with a frequency > 1, or reporting an intensity of 3 combined with a frequency > 3, and (B) meeting criterion A for ≥ 5 of the 10 items. All other response combinations were considered to indicate “not high” moral distress. This reflects the constructs definition by capturing high moral distress as an accumulated burden of highly disturbing, recurrent morally challenging situations.

Applying these two criteria resulted in a proportion of high moral distress of 28% (*n* = 259) among the respondents. Stratifying by occupation revealed a statistically significant higher proportion of high moral distress (*p* = 0.003) among midwives (33%) and registered nurses (31%) compared to physicians (22%) and assistant nurses (21%).

The proportion of respondents experiencing a high intensity and a high frequency for individual items (criteria A) ranged from 16% to 43%. It was more common to exceed criteria A on items being related to not being able to provide adequate care due to budgetary cuts (42%), or staff shortages (43%), while it was more uncommon to experience that identified moral problems were not addressed properly (17%), or to take decisions with limited knowledge (17%). Overall, 21% of the respondents did not exceed criteria A for any single item, while a majority (51%) exceeded the criteria on ≥ 3 individual items. The proportion of maternity and neonatal healthcare workers experiencing high moral distress, and the frequency of exceeding individual items, were consistent with the results from the interviews and the assessments made by the expert group.

*" If several [of the midwives] are also juniors… and then myself.*

*Then it’s really tough. Yeah*,* it’s not fun “*.

*                                                                                      -Physician, 1 year work experience, interview 5*

### Construct validity of the Swedish maternal and neonatal version of MDS-R

**Fig. 1:**
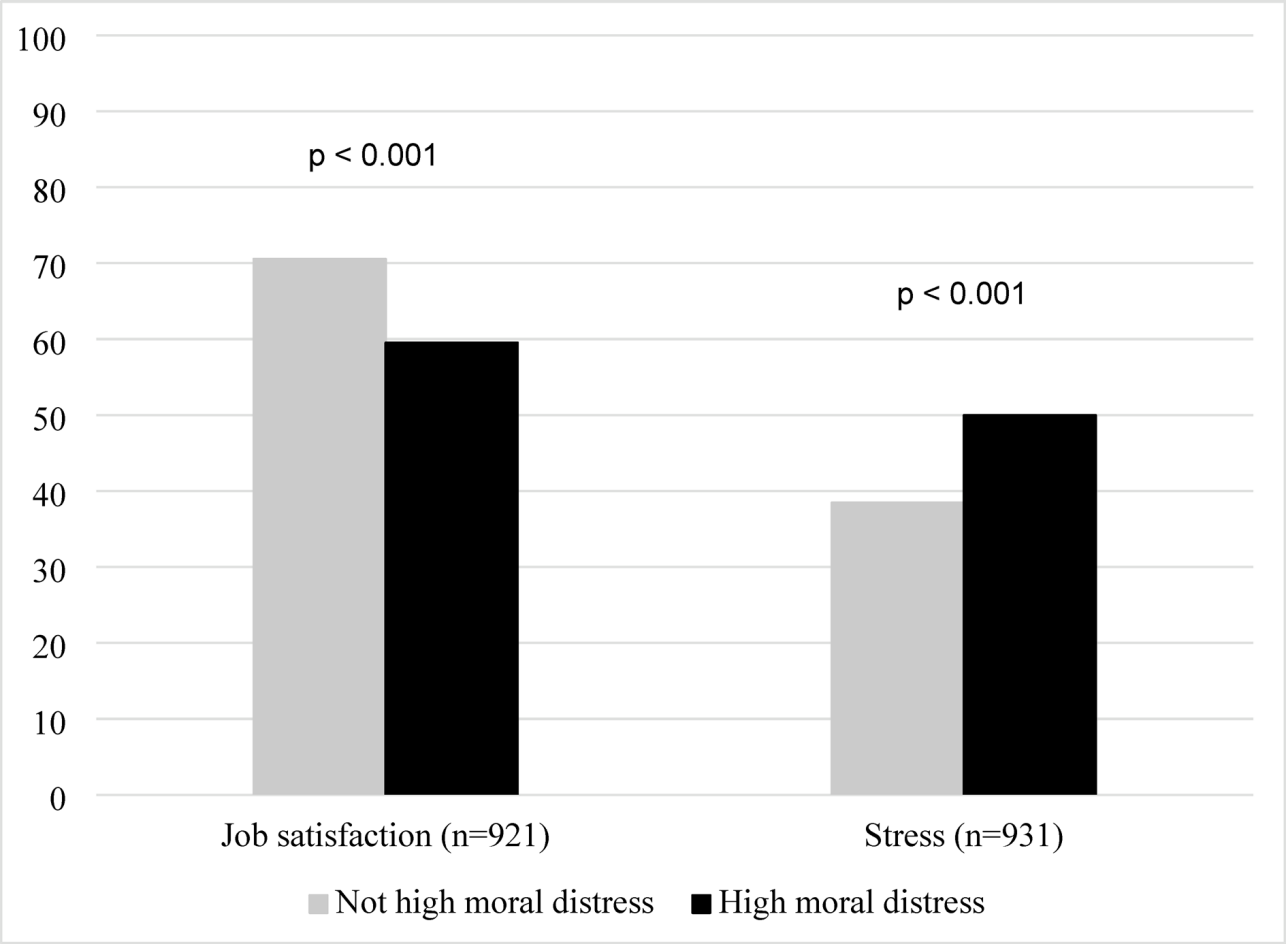
Levels of job satisfaction and stress stratified by the presence or absence of high moral distress (*p* < 0.001, respectively).

Having high moral distress was associated with lower job satisfaction (U = -7.6877, *p* < 0.001, *r*=-0.25) and higher levels of stress (U = 6.7156, *p* < 0.001, *r* = 0.22) compared to not having high moral distress (Fig. 1).

**Fig. 2:**
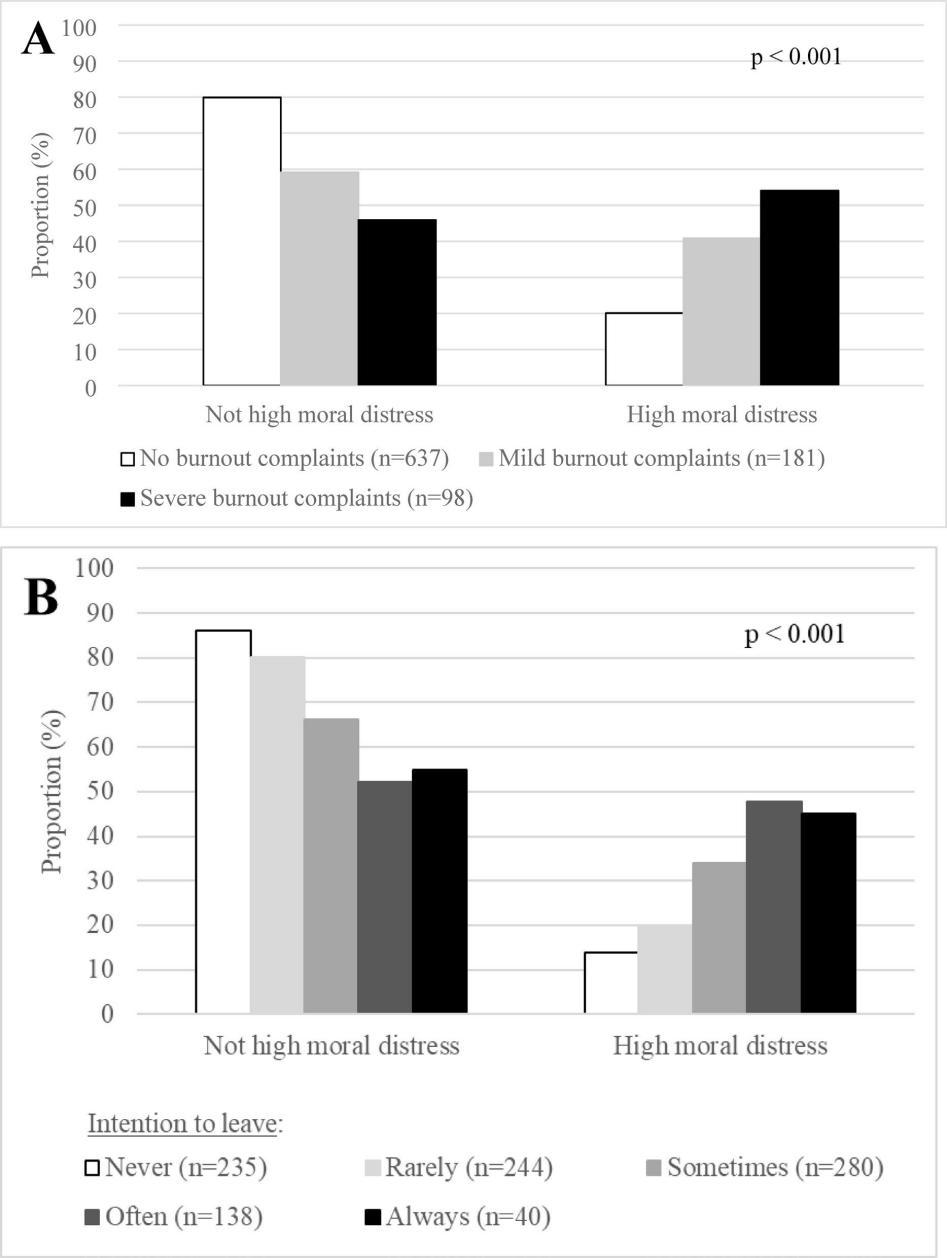
Distribution of respondents experiencing none, mild and severe burnout complaints (panel **A**), and never, rarely, sometimes, often and always reporting an intention to leave (panel **B**), stratified by the presence or absence of high moral distress.

Furthermore, the proportion of respondents with mild and severe burnout complaints, and respondents with intentions to leave, was higher among respondents with high moral distress compared to those without high moral distress (H(3) = 78.3504, *p* < 0.001, ŋ^2^=0.08 and H(5) = 69.0511, *p* < 0.001, ŋ^2^=0.07, Fig. 2). About 21% of the respondents with high moral distress experienced severe burnout complaints and another 29% experienced mild burnout complaints, compared with 7% and 16% among those not having high moral distress. Similarly, 32% of the respondents with high moral distress always or often had intentions to leave, compared to 14% of those not having high moral distress. Meanwhile, 19% of the respondents with high moral distress never or rarely reported intentions to leave, compared to 59% of respondents not having high moral distress (Fig. 2).

## Discussions

The initial testing of the Swedish maternal and neonatal version of the MDS-R indicates promising evidence of content, face, and construct validity. Consequently, the adapted version may be used to investigate the intensity and frequency of moral distress with the aim of increasing knowledge of the occurrence, and prevention of moral distress within the Swedish maternal and neonatal healthcare context. We have also presented a new criterion-based index for high moral distress that may be used to identify individuals with high moral distress. Using this index in a national sample revealed that almost one-third of maternal and neonatal healthcare workers in Sweden experienced high moral distress. In addition, high moral distress was related to decreased job satisfaction and increased levels of stress, burnout complaints, and intentions to leave, which highlights the need for evidence-based interventions aimed at to decrease moral distress within the maternal and neonatal healthcare sector to secure a sustainable and healthy workforce. Because the adapted MDS-R was treated as a formative measurement model, traditional psychometric evaluations (e.g., internal consistency, factor structure) are not applicable. The present study therefore provides validation evidence relevant to formative constructs, i.e. content and face validity, criterion-based index development, and its construct validity, rather than a comprehensive psychometric validation based on reflective measurement assumptions. Future studies should further evaluate the stability and reproducibility of the high moral distress classification over time, including cross-validation in other samples/settings.

When assessing the content and face validity, particular attention was given to limiting bias from conceptual ambiguities and known confounders by distinguishing between exposure to morally challenging situations (stressors) and experiencing moral distress^10,11^. This distinction is well established in traditional stress theories, such as Lazarus and Folkman’s transactional model of stress and coping, which conceptualises stress as a psychological response arising from the cognitive appraisal of a stressor rather than the stressor itself^29^. Further, Conservation of Resources (COR) theory emphasises that stress occurs when valued resources are threatened, lost, or insufficient to meet demands, framing stress as a consequence of resource dynamics rather than mere exposure to challenging events^30,31^. According to both theories, stress is conceptualised as a dynamic process arising from the interaction between perceived demands and coping resources, influenced by cognitive appraisal mechanisms and actual or threatened resource loss. Applying these theoretical perspectives ensures that the measurement of moral distress captures the subjective experience of resource loss and appraisal processes. The use of cognitive interviews allowed us to gain insight into how the intended respondents interpreted the adapted items. The distinction between morally challenging situations and moral distress was believed to be captured by separately assessing the intensity and frequency. However, the use of separate assessments may lead to respondents being asked to assess the intensity of a morally challenging situation that has not occurred. This type of hypothetical thinking may introduce response bias if the respondent becomes uncertain about how to answer, which has previously been described^32^. This was, however, not indicated in our study, which may be explained by the fact that all items were regarded as relevant, and the respondents could operationalise each statement into morally challenging situations. In addition, af Sandberg et al. changed the order of these two questions, in the Swedish paediatric version of the MDS-R, on which we based our version, in relation to the original MDS-R, to limit the negative effect of hypothetical thinking^16^.

Another challenge in the past has been to distinguish morally challenging situations from non-morally challenging situations in quantitative measurement of moral distress^10,11^. Limiting this bias has been more difficult, since items within the instrument contain both perspectives, as pointed out by Oelhafen et al.^11^. However, the cognitive interviews indicated that respondents were highly focused on the potential negative effects on patient safety and quality of care, which may be partly explained by the high work engagement and professional identity within maternity and neonatal care^33,34^.

However, it is important to point out that failing to distinguish between these types of situations will not prevent the ability to capture moral distress, rather, moral distress will not be captured in isolation^11^. Thus, to disentangle moral distress from distress caused by adverse working conditions themselves requires study designs that measure both morally challenging situations and working conditions, using multivariable analytical approaches to simultaneously assess their combined effects (Oelhafen et al. 2025).

The use of a criterion-based index for high moral distress is both in line with recommendations for handling formative measurement models^17^ and may potentially reduce the challenge of interpreting the index, which has been identified in indices measuring total moral distress that use conventional methods^11^. Using this criterion-based index revealed that 28% of maternal and neonatal healthcare workers experienced high moral distress. Prevalence rates of moral distress are hard to compare between studies and contexts due to the use of different instruments and definitions^10,11^, but the proportion of high moral distress was in agreement with a priori assessments made by the expert group. Furthermore, Swedish maternity and neonatal healthcare has been under pressure for a long time^24,35,36^, and there is a shortage of specialist-trained professionals. Moral distress related to a shortage of staff was also the item with the highest proportion of respondents exceeding the criteria for high moral distress in a single item. When stratified by occupations, it was more common to experience high moral distress among midwives and registered nurses compared to physicians and assistant nurses. The difference between registered nurses and physicians has been seen in other studies, which may reflect the differences in power hierarchy between these groups^14,37^. The lower proportion of high moral distress among assistant nurses could reflect differences in decision-making between assistant nurses compared with the other professions, which is one of the areas surveyed in the MDS-R, but further studies are needed to disentangle potential differences in moral distress between professions.

As hypothesised, high moral distress was also associated with decreased job satisfaction and increased levels of stress, burnout complaints, and intention to leave, which strengthens the evidence for the construct validity of the adapted version of the MDS-R in the context of Swedish maternal and neonatal healthcare workers. Similar associations have also been observed in previous, predominantly cross‑sectional, studies and longitudinal studies examining temporal or reciprocal relationships remain scarce, especially within the maternal and neonatal care^1,4–6^. Naturally, high moral distress cannot fully explain job satisfaction, stress, burnout complaints, and intentions to leave, since employee health and intentions to leave are multifaceted challenges affected by many factors at different levels within the organisation^11,38^. Nevertheless, the observed differences between individuals with and without high moral distress were substantial and considered meaningful. The results are aligned with previous research, were changes in job satisfaction and stress levels were observed among healthcare workers facing the greatest workload increases during the pandemic^24^. For burnout complaints, individuals with high moral distress reported severe burnout complaints about three times more frequently compared to those with low moral distress. Due to the cross‑sectional design, the study can identify associations but cannot determine their direction, and burnout may therefore affect the experience of moral distress rather than the reverse. Future research employing longitudinal designs is needed to clarify these temporal and potentially reciprocal relationships. A similar pattern was observed for intentions to leave. The levels of burnout complaints found among individuals without high moral distress in this study correspond to levels found in other Swedish populations within maternal and neonatal healthcare^36,39,40^.

### Strengths and limitations

A strength of this study was not only focus on different validation aspects but also its emphasis on the practical usability of the instrument by defining an expert-developed cut-off for high moral distress that can be used for targeted interventions. We also employed an index calculation method that better aligns with the structure of the MDS-R, which we consider to represent a formative measurement model. As such, the traditional approach to calculating mean scores, based on the assumption of unidimensionality, is not appropriate. Another strength was the use of cognitive interviews, which has the potential to reveal additional problems, such as issues with question design or usability that might otherwise have been overlooked. A limitation was the limited number of respondents in the cognitive interviews, which may affect the transferability of the findings. To limit this potential bias, experienced respondents were purposively sampled to gain a high information power^20^. These participants had up to 36 years of experience, were currently employed in a wide range of units across two different organisations, and most of them had experience working across other units throughout the years. The cross‑sectional design prevented causal inferences, and future research employing longitudinal designs is needed to clarify the temporal and potentially reciprocal relationships between moral distress, burnout, and stress. In addition, the present validation work has defined boundaries that should be acknowledged. The instrument underwent substantial adaptation, which may affect content equivalence with the original MDS‑R and limits direct comparability with studies using earlier versions. Moreover, several measurement properties typically evaluated in reflective models, such as internal consistency, factor structure, and measurement invariance, were not assessed, as they are not appropriate for a formative conceptualisation. Finally, the proposed cut‑off for high moral distress is inherently normative, as it is based on expert judgement rather than empirical outcome‑based calibration, highlighting the need for further studies to examine its generalisability, empirical grounding, and usefulness across different occupational groups and contexts.

## Conclusions

The results indicate that this adapted version of the MDS-R questionnaire has the potential to be used as a tool for systematically identifying individuals at risk of moral distress and for increasing knowledge of how adverse effects on employee health due to moral distress may be prevented within Swedish maternal and neonatal healthcare. The findings from the study further support the relationship between high moral distress and adverse effects on job satisfaction, stress, burnout complaints, and intention to leave, and highlight the emerging need to address moral distress within the maternal and neonatal healthcare sector.

## Supplementary Information

Below is the link to the electronic supplementary material.

Supplementary Material 1

## Data Availability

The datasets generated during and/or analysed during the current study are available from the corresponding author on reasonable request.
