# Supplementary material for: Measuring moral distress in Swedish maternal and neonatal healthcare: validation of an adapted MDS‑R and development of a criterion‑based index
Source: Sci Rep. 2026 May 12;16:14763. doi: 10.1038/s41598-026-52337-6 (PMC13168699; doi:10.1038/s41598-026-52337-6)
Supplement: Supplementary file 1 — Supplementary Material 1 [file 41598_2026_52337_MOESM1_ESM.pdf]

# Interview Guide – Cognitive Interviews MDS-R

1. **What is your profession?**
2. **What is your age?**
3. **How many years have you worked in your profession?**
4. **Think aloud** – Read each question about *"How would this situation affect you"* out loud. Ask the person to think aloud while answering.
    - How do they understand the question?
    - Can they rephrase it in their own words?
    - What associations do they make when responding?
    - Does the question feel relevant?
5. **How do the response options work?**
    - What characterizes *"not at all"* versus *"very negatively"*?
    - What reasoning did they use when answering?
    - How would they have answered if the situation had never occurred?
6. **Now we focus on** *"How often have you experienced this situation?"*
    - How do you understand that question?
    - What would happen if it had never happened to you—how would you respond then?
    - Was there any situation you've never experienced?
7. **How do you reason about the scale from** *"Never"* to *"Very often"*?
    - How frequently must something occur for you to consider it *"very often"*?
8. **If we group the responses** – Which number(s) between 0–4 for *"not at all"* versus *"very negatively"* represent a high intensity of moral stress in your opinion?
9. **If we group the responses** – Which number(s) between 0–4 for *"never"* versus *"very often"* represent a high frequency of moral stress in your opinion?
10. **How do you evaluate the relationship between** high intensity with low frequency versus low intensity with high frequency of moral stress?
11. **Are there any situations you feel are missing?**
12. **Anything else you'd like to add or think of regarding the questionnaire?**
